# The Amplifying Effect of Conflicts on Case Fatality Rate of COVID-19: Evidence From 120 Countries

**DOI:** 10.3389/fpubh.2021.681604

**Published:** 2021-08-04

**Authors:** Yonghui Zhai, Dayang Jiang, Giray Gozgor, Eunho Cho

**Affiliations:** ^1^School of Business, Henan Normal University, Xinxiang, China; ^2^School of Economics, Tianjin University of Commerce, Tianjin, China; ^3^Faculty of Political Sciences, Istanbul Medeniyet University, Istanbul, Turkey; ^4^Accounting and Finance Department, North Carolina Agricultural and Technical State University, Greensboro, NC, United States

**Keywords:** COVID-19 pandemic, case fatality rate, mortality risk, armed conflicts, machine learning estimator

## Abstract

Using the COVID-19 database of Johns Hopkins University, this study examines the determinants of the case fatality rate of COVID-19. We consider various potential determinants of the mortality risk of COVID-19 in 120 countries. The Ordinary Least Squares (OLS) and the Kernel-based Regularized Least Squares (KRLS) estimations show that internal and external conflicts are positively related to the case fatality rates. This evidence is robust to the exclusion of countries across different regions. Thus, the evidence indicates that conflict may explain significant differences in the case fatality rate of COVID-19 across countries.

## Introduction

The COVID-19 pandemic has affected every aspect of the economy and society. Since this new type of coronavirus is significantly more fatal than the common flu and is transmittable from one human to another, the pandemic has quickly spread across the globe. In mid-May 2021, there were 168 million infected people and almost 3.4 million had died worldwide (1); however, the case fatality rate (henceforth CFR), i.e., the total deaths relative to total cases, differs across countries. For example, according to the COVID-19 database of Johns Hopkins University provided by Dong et al. (1), on May 15, 2021, there were 33,715,951 COVID-19 cases and 600,147 deaths in the United States, for a CFR of 1.78%. This CFR is lower than the CFR of the world, which was 2.07% on that date. Interestingly, there were 4,450,777 total cases and 127,679 total deaths due to the COVID-19 pandemic in the United Kingdom as of May 15, 2021, with a CFR of 2.86%. The data from the database of Johns Hopkins University, on May 15, 2021, show that the CFR of the top 20 most infected countries in the world has changed from 0.87 (Turkey) to 9.25 (Mexico).

Conflicts are a significant reflection of political instability, which can provide a better ground for COVID-19 transmission. A higher number of infected people implies a higher number of deaths. Conflicts should be positively correlated with less effective testing and tracing policy, meaning that more infection cases might go undetected, biasing the CFR toward higher estimates. Therefore, the CFR might be higher in certain countries despite similar infection dynamics. For instance, internal protests and similar social movement events, such as the 2020 Black Lives Matter protests and the 2021 storming of the United States Capitol Building, can increase the CFR of COVID-19 (2). Conflicts affect CFR because of increased transmission, thereby increasing the death probability (3).

Given this context, this study examines the determinants of CFRs related to COVID-19 across 120 countries. There are previous studies that focused on the determinants of CFRs of COVID-19. For instance, Banik et al. (4) and Khan et al. (5) found that the public health system and age structure are the main determinants of the CFR. Similarly, Moosa and Khatatbeh (6) found that age structure and population density are the main determinants of the CFR. Daw (7) showed that armed conflict is the main determinant of the spread of COVID-19 in Libya, Syria, and Yemen. Elgar et al. (8) indicated that social capital and income inequality are the main drivers of COVID-19 deaths in 84 countries. Finally, Sorci et al. (9) observed that share of the population over 70, income per capita, and democracy influence the CFR of a country.

Unlike previous studies, this study implements the Kernel-based Regularized Least Squares (KRLS) to address potential issues of non-linearity and multicollinearity. In doing so, this study obtains evidence on the direction, sign, and magnitude of the causality between armed conflict and COVID-19. In addition, we observe that conflicts positively affect CFR because of increased transmission and increasing death probability.

The rest of the study is organized as follows. First, the paper explains empirical strategy and data. Then, it discusses empirical results and provides robustness checks and concludes.

## Empirical Strategy, Estimation Procedure, and Data

### The Baseline Empirical Model and Estimation Procedure

This study estimates the following equation:

(1) $$\begin{array}{r} CFR_{i}=\gamma_{0}+{\gamma_{1}\;X}_{i}{+\varepsilon }_{i,t} \end{array}$$

*CFR*_*i*_ is the case fatality rate in country i; *X*_*i*_ is a vector of control variables, which will be explained accordingly; and ε_*i*_ indicates the error term in the estimations. The empirical examination is based on a recently available cross-sectional dataset for 120 countries, and the list of countries in the dataset is provided in Appendix Table I. The dataset includes countries at very different developmental stages, explaining cross-country differences in the CFR.

We estimate Equation (1) using the Ordinary Least Squares (OLS) estimator, the traditional method. At this stage, we also consider the KRLS, a machine learning method defined in Hainmueller and Hazlett (10). The KRLS estimator can learn the data to decrease potential misspecification bias. The KRLS method can also solve potential problems of non-linearity and multicollinearity in the OLS estimations (10).

### Data

#### Dependent Variable

The dependent variable is the CFR, and it is calculated as the number of total COVID-19 deaths relative to the number of the total confirmed COVID-19 cases, which is accessed on March 15, 2021. It is the benchmark measure of the fatality rate of the mortality risks of COVID-19 in the empirical epidemiology literature (11). In addition, the CFR shows the likelihood of death in the context of an ongoing infection (12), and the CFR indicator is calculated using the data in Dong et al. (1).

#### Control Variables

Following previous articles, we focus on various potential drivers of the CFR of COVID-19. Finally, we explain the control variables as follows:

##### Demographics and Social Networks

The CFR of COVID-19 is highly related to age. Older people are expected to have higher mortality due to the COVID-19. Therefore, the share of people aged 65 and above in the total population and those aged between 0 and 14 in the total population are added. Population density (in logarithmic form) also captures the degree of urbanization and social networks.

##### Health Capacity, Testing, and Tracking

Health equipment (hospital beds) per 1,000 people and physicians per 1,000 people capture health infrastructure conditions. These indicators are drawn from World Bank data (13). Testing and tracking policies (index from 0 to 5, with a higher level of the index indicating higher quality testing and tracking policies) are determinants of the CFR of COVID-19. This indicator is drawn from the work of Hale et al. (14).

##### Climate

average temperatures can affect the CFR of COVID-19 if the spread of the virus is similar to that of the seasonal flu. Average temperature data (in degrees Celsius) are downloaded from the website of the World Bank (15).

##### Economic Structure

The economic complexity index captures the effects of economic development on the CFR (a higher level on the index indicates a higher economic development level). Related data are obtained from Hausmann et al. (16). Following Gozgor and Ranjan (17), the post-tax Gini index of income inequality (index from 0 to 1, with a higher index level indicating greater income inequality) is also included. Related data are obtained from the Standardized World Income Inequality Database (SWIID) (version 9.1) of Solt (18). The index of labor market regulations (index from 0 to 10, with a higher index level indicating greater labor market flexibility) is also included since it captures unemployment payments, minimum wages, and working conditions. These data are downloaded from the economic freedom dataset of Gwartney et al. (19). We suggest that economic structure measured by economic complexity, income inequality, and labor market regulations can affect the CFR of COVID-19.

##### Globalization

It can affect the CFR of COVID-19 (20). Therefore, the overall KOF globalization index in logarithmic form (index from 0 to 100, with a higher index level indicating greater globalization) is included in the estimations. The data are accessed from the revised KOF globalization dataset of Gygli et al. (21). In addition, refer to Dreher (22) and Gozgor (23) for the methodology used in the KOF globalization indices.

##### Institutional Quality

Institutions with higher quality can control COVID-19 more efficiently. For this purpose, we use the democracy/autocracy spectrum measured by the Polity2 (index from −10 to 10, with −10 indicating full autocracy and 10 indicating full democracy) is added to the estimations. This indicator is obtained from the Polity V annual time series proposed by Marshall and Gurr (24).

##### Conflicts and Political Instability

Conflicts are defined by the systematic and sustained use of lethal violence by organized groups that result in at least 500 directly related deaths throughout the episode. Internal and external conflicts are an index from 0 to 14. A higher level of the index indicates heavier armed conflicts. This index captures the events related to international violence, international warfare, international independence war, civil violence, civil warfare, ethnic violence, and ethnic warfare, which can significantly affect the CFR of COVID-19. The related data are obtained from the major episodes of political violence dataset of Marshall (25).

Finally, all indicators and the summary of the descriptive statistics are provided in Table 1.

**Table 1:** Descriptive statistics.

**Indicator**	**Definition**	**Data source**	**Mean**	**SD**	**Min**.	**Max**.	**Obs**.
Dependent variable: case fatality rate	Level	Dong et al. (1)	2.302	2.801	0.050	28.91	120
Demographics: share of population: ages 65 and above	%	World Bank (13)	9.865	6.570	1.144	27.04	120
Demographics: share of population: ages 0–14	%	World Bank (13)	25.69	10.13	12.33	47.30	120
Social networks: population density	Logarithmic form	World Bank (13)	4.228	1.284	0.683	8.976	120
Health capacity: health equipment: hospital beds	Per 1,000 people	World Bank (13)	3.044	2.540	0.001	13.05	120
Health capacity: physicians	Per 1,000 people	World Bank (13)	2.146	1.603	0.014	7.120	120
Testing and tracking policies	Index from 0 to 5	Hale et al. (14)	3.241	1.276	0.000	5.000	120
Climate: average temperatures	Level in celsius degrees	World Bank (15)	17.14	8.606	−5.350	28.25	120
Economic structure: economic complexity	Level in index	Hausmann et al. (16)	0.021	1.007	−2.008	2.309	120
Economic structure: post-tax Gini	Index from 0 to 1	Solt (18)	0.380	0.075	0.227	0.625	120
Economic structure: labor market regulations	Index from 0 to 10	Gwartney et al. (19)	6.298	1.265	2.24	8.98	120
Globalization: KOF globalization index	Log Index from 0 to 100	Dreher (22) & Gygli et al. (21)	4.201	0.193	3.756	4.508	120
Institutional quality: democracy/autocracy spectrum	Index from −10 to 10	Marshall and Gurr (24)	4.741	5.995	−10.00	10.00	120
Conflicts: sum of internal and external conflicts	Index from 0 to 14	Marshall (25)	0.400	1.140	0.000	6.000	120

## Empirical Findings and Robustness Checks

### Ordinary Least Squares and KRLS Findings

Table 2 reports the findings of empirical estimations. Colum (I) provides the results of the OLS estimations, and Column (II) provides the findings of the KRLS estimations. The dependent variable is the CFR in 120 countries in both estimations.

**Table 2:** Results of Ordinary Least Squares (OLS) and Kernel-based Regularized Least Squares (KRLS) estimations (all countries).

**Indicator**	**OLS (I)**	**KRLS (II)**
Share of population: ages 65 and above	0.075 (0.096)	0.005 (0.006)
Share of population: ages 0–14	−0.020 (0.057)	−0.001 (0.004)
Log population density	−0.197 (0.221)	−0.036 (0.057)
Health equipment	−0.121 (0.146)	−0.010 (0.022)
Physicians	−0.077 (0.285)	−0.018 (0.036)
Average temperatures	−0.003 (0.005)	−0.001 (0.006)
Testing and tracking policies	−0.245 (0.213)	−0.091 (0.058)
Economic complexity	0.529 (0.530)	0.021 (0.055)
Post-tax Gini	0.774 (4.909)	0.004 (0.865)
Labor market regulations	−0.039 (0.209)	−0.046 (0.059)
Log globalization	−5.273 (3.747)	−0.281 (0.246)
Democracy/autocracy spectrum	0.031 (0.056)	0.008 (0.010)
Internal- and external conflicts	0.775^***^ (0.233)	0.167^***^ (0.045)
R-squared	0.1819	0.2093
Observations	120	120

*The dependent variable is the case fatality rate (CFR). The robust standard errors are in ()*.

*** *p < 0.01*.

In both the OLS and KRLS estimations, all variables are insignificant except for internal and external conflicts. Specifically, a greater share of people aged 65 and above in the total population increases the CFR. Conversely, a greater share of people aged between 0 and 14 in the total population decreases the CFR as shown in Table 3. This evidence is in line with the intuition that COVID-19 mainly kills older people, and young people have a lower CFR (6). Thus, population density is negatively associated with the CFR of COVID-19. In addition, previous studies suggested that population density leads to higher transmission risks of COVID-19 [see (26)]; however, there is mixed evidence on the effects of population density on the CFR of COVID-19. For instance, Khan et al. (5) show that population density has insignificant effects on the CFR of COVID-19 in countries, particularly those with a lower per capita income, where lockdowns and social distancing have been applied.

**Table 3:** Sensitivity analyses of the KRLS results.

**Sensitivity analysis: excluding**	**OECD**	**Europe**	**Asia**	**Africa**	**South America**	**Central & North America**
**Indicator**	**(I)**	**(II)**	**(III)**	**(IV)**	**(V)**	**(VI)**
Share of population: ages 65 and above	0.003 (0.008)	0.005 (0.009)	0.008 (0.044)	0.003 (0.004)	0.005 (0.006)	0.008 (0.015)
Share of population: ages 0–14	−0.002 (0.005)	−0.003 (0.005)	−0.002 (0.003)	−0.002 (0.005)	−0.001 (0.004)	−0.005 (0.011)
Log population density	−0.034 (0.054)	−0.044 (0.051)	−0.004 (0.005)	−0.019 (0.036)	−0.027 (0.059)	−0.006 (0.014)
Health equipment	−0.017 (0.020)	−0.023 (0.021)	−0.002 (0.018)	−0.001 (0.015)	−0.007 (0.023)	−0.002 (0.005)
Physicians	−0.015 (0.035)	−0.013 (0.038)	−0.001 (0.002)	−0.012 (0.028)	−0.023 (0.036)	−0.005 (0.008)
Average temperatures	−0.004 (0.006)	−0.004 (0.008)	−0.001 (0.004)	−0.001 (0.004)	−0.001 (0.007)	−0.001 (0.001)
Testing and tracking policies	−0.072 (0.053)	−0.078 (0.057)	−0.006 (0.005)	−0.049 (0.037)	−0.090 (0.060)	−0.021 (0.014)
Economic complexity	0.024 (0.077)	0.030 (0.064)	0.009 (0.039)	0.011 (0.038)	0.039 (0.056)	0.002 (0.012)
Post-tax Gini	0.457 (1.001)	0.539 (1.160)	0.033 (0.057)	0.287 (0.585)	0.171 (0.910)	0.019 (0.208)
Labor market regulations	−0.040 (0.058)	−0.041 (0.057)	−0.005 (0.004)	−0.016 (0.039)	−0.044 (0.068)	−0.009 (0.015)
Log globalization	−0.473 (0.341)	−0.467 (0.340)	−0.003 (0.016)	−0.224 (0.178)	−0.316 (0.252)	−0.080 (0.057)
Democracy/autocracy spectrum	0.002 (0.011)	0.004 (0.011)	0.002 (0.009)	0.004 (0.005)	0.005 (0.011)	0.001 (0.002)
Internal- and external conflicts	0.153^***^ (0.041)	0.177^***^ (0.045)	0.006^**^ (0.002)	0.057^**^ (0.026)	0.194^***^ (0.048)	0.031^***^ (0.010)
R-squared	0.1841	0.1846	0.2719	0.1447	0.2296	0.0487
Observations	86	86	85	91	110	108

*The dependent variable is the case fatality rate (CFR). The robust standard errors are in ()*.

*** *p < 0.01 and*

** *p < 0.05*.

Healthcare indicators are also included in both estimations. For example, greater healthcare supplies, higher numbers of physicians, and more robust testing and tracking policies negatively affect the CFR of COVID-19; however, their coefficients are statistically insignificant. These results are in line with previous findings, such as those of Banik et al. (4), Khan et al. (5), Moosa and Khatatbeh (6), and Sorci et al. (9). We also find no evidence of a pattern between the CFR of COVID-19 and average temperatures. Again, this evidence aligns with Jamil et al. (27), implying that COVID-19 does not behave like the seasonal flu.

We observe that economic complexity increases the CFR of COVID-19. Given that economic complexity is highly positively correlated with per capita income, this evidence is in line with findings presented in previous studies. Furthermore, greater income inequality, measured by post-tax Gini coefficients, increases the CFR of COVID-19. Elgar et al. (8) indicated that relative income differences in a country could also affect infectious disease patterns. Indeed, income inequality is a strong indicator of inequality in healthcare access, increasing the CFR of COVID-19. In addition, greater labor market flexibility decreases the CFR of COVID-19. Labor market flexibility is, in general, positively associated with the ease of adopting work-from-home arrangements, which decreases the risks of virus exposure (28). We also find that a higher level of globalization reduces the CFR of COVID-19. It is suggested that globalization leads to higher transmission risks of COVID-19 [refer to, e.g., (9)]; however, globalization (especially social globalization indicators, such as internet access) promotes the effects of lockdowns on the spread of COVID-19. Economic globalization can also promote connection with the rest of the world, resulting in greater information sharing and technology to combat COVID-19 (20). The democracy/autocracy spectrum, measured by the Polity2 index, shows that more democratic societies have higher levels of COVID-19 related CFR. This evidence is in line with Sorci et al. (9). It may be related to the issue that it is more difficult to implement hard lockdowns and social distancing in more democratic societies.

The main finding of this study is that internal and external conflicts are positively related to the CFR of COVID-19. Evidence for this finding is observed when the OLS and the KRLS estimations are utilized. The estimated coefficients of total conflicts are around 0.775 and 0.167 in the OLS and the KRLS estimations. Furthermore, both coefficients are statistically significant at the 1% level. Finally, the R-squared of the KRLS estimations is higher than the OLS estimations. This evidence indicates that the KRLS has more explanatory power than the OLS estimations.

### Robustness Checks

Table 3 also provides several sensitivity analyses to check the validity of the baseline KRLS estimations. Specifically, following the spirit of Jha and Gozgor (29), we exclude (i) Organization for Economic Co-operation and Development (OECD) member countries; (ii) European countries; (iii) Asian countries; (iv) African countries; (v) South American counties; (vi) Central and North American countries. The related results are provided in Columns (I), (II), (III), (IV), (V), and (VI) of Table 3, respectively.

In all the KRLS estimations, the only statistically significant coefficients are internal and external conflicts. Specifically, the greater the share of the population aged 65 and above the higher the CFR of COVID-19, while the greater the share of the population aged between 0 and 14 the lower the CFR. Population density is negatively related to the CFR of COVID-19. Greater supplies of health equipment, higher numbers of physicians, and more robust testing, and tracking policies negatively affect the CFR of COVID-19. There is no evidence of a pattern between the CFR of COVID-19 and average temperatures. Economic complexity increases the CFR of COVID-19. Income inequality increases the CFR of COVID-19. In addition, a greater labor market flexibility decreases the CFR of COVID-19, and a higher level of globalization reduces the CFR of COVID-19. Finally, the more democratic a society, as measured along the democracy/autocracy spectrum, the higher the CFR of COVID-19.

The main and most robust finding in this study is that internal and external conflicts are associated with the CFR of COVID-19. This evidence is still valid when the countries on different continents are excluded. The estimated coefficients of the internal and external conflicts indicator are between 0.006 and 0.194. Thus, all coefficients are statistically significant at the 5% level at least.

## Conclusion

This study investigates the potential drivers of the CFR of COVID-19 across 120 countries. The results of the OLS and the KRLS estimators indicate that internal and external conflicts are positively associated with the CFR. Other potential determinants are not robust to different estimators. Our main evidence is robust to excluding countries across regions, i.e., the OECD, Europe, Asia, Africa, South America, and Central and North America. Therefore, we suggest that conflicts are significant factors explaining why there are significant differences in the CFR across developed and developing economies.

Overall, monitoring internal and external conflicts may help identify the CFR of COVID-19. These results suggest that the mortality risk of COVID-19 will increase because of the escalation in conflicts. Our findings are consistent with the recent study by Daw (7) that links higher armed conflict levels to the greater spread of the COVID-19 pandemic in Libya, Syria, and Yemen. Future articles can use different indicators and econometric techniques using survey data to predict the drivers of the spread of the COVID-19 pandemic and the CFR in different countries.

## Data Availability Statement

The datasets presented in this study can be found in online repositories. The names of the repository/repositories and accession number(s) can be found in the article/supplementary material.

## Author Contributions

YZ: writing original draft & methodology. DJ: writing original draft & estimations. GG: writing original draft & data collection. EC: reviewing original draft & project management. All authors contributed to the article and approved the submitted version.

## Conflict of Interest

The authors declare that the research was conducted in the absence of any commercial or financial relationships that could be construed as a potential conflict of interest.

## Publisher's Note

All claims expressed in this article are solely those of the authors and do not necessarily represent those of their affiliated organizations, or those of the publisher, the editors and the reviewers. Any product that may be evaluated in this article, or claim that may be made by its manufacturer, is not guaranteed or endorsed by the publisher.

## Appendix

**Appendix Table 1:** The 120 countries in the dataset.

Albania, Algeria, Angola, Argentina, Australia, Austria, Azerbaijan, Bangladesh, Belarus, Bolivia, Bosnia and Herzegovina, Brazil, Bulgaria, Cambodia, Cameroon, Canada, Chile, China, Colombia, Congo Republic, Costa Rica, Cote d'Ivoire, Croatia, Czech Republic, Denmark, Dominican Republic, Ecuador, Egypt, El Salvador, Estonia, Ethiopia, Finland, France, Gabon, Georgia, Germany, Ghana, Greece, Guatemala, Guinea, Honduras, Hungary, India, Indonesia, Iran, Ireland, Israel, Italy, Jamaica, Japan, Jordan, Kazakhstan, Kenya, Korea Republic, Kuwait, Kyrgyzstan, Laos, Latvia, Lebanon, Liberia, Libya, Lithuania, Madagascar, Malawi, Malaysia, Mali, Mauritania, Mauritius, Mexico, Moldova, Mongolia, Morocco, Mozambique, Netherlands, New Zealand, Nicaragua, Nigeria, North Macedonia, Norway, Oman, Pakistan, Panama, Papua New Guinea, Paraguay, Peru, Philippines, Poland, Portugal, Qatar, Romania, Russia, Saudi Arabia, Senegal, Serbia, Singapore, Slovakia, Slovenia, South Africa, Spain, Sri Lanka, Sudan, Sweden, Switzerland, Tajikistan, Tanzania, Thailand, Trinidad and Tobago, Tunisia, Turkey, Uganda, Ukraine, United Arab Emirates, United Kingdom, United States, Uruguay, Venezuela, Vietnam, Yemen, Zambia, and Zimbabwe.
